# Use of MIRUS™ for MAC-driven application of isoflurane, sevoflurane, and desflurane in postoperative ICU patients: a randomized controlled trial

**DOI:** 10.1186/s13613-019-0594-8

**Published:** 2019-10-16

**Authors:** Martin Bellgardt, Adrian Iustin Georgevici, Mitja Klutzny, Dominik Drees, Andreas Meiser, Philipp Gude, Heike Vogelsang, Thomas Peter Weber, Jennifer Herzog-Niescery

**Affiliations:** 1 Department of Anaesthesiology and Intensive Care Medicine, Ruhr-University Bochum, St. Josef Hospital, Gudrunstraße 56, 44791 Bochum, Germany; 2 Department of Anaesthesiology, Intensive Care Medicine and Pain Medicine, Saarland University Medical Center, Homburg/Saar, Germany

**Keywords:** Isoflurane, Sevoflurane, Desflurane, Inhalational sedation, MAC-driven sedation

## Abstract

**Background:**

The MIRUS™ (TIM, Koblenz, Germany) is an electronical gas delivery system, which offers an automated MAC (minimal alveolar concentration)-driven application of isoflurane, sevoflurane, or desflurane, and can be used for sedation in the intensive care unit. We investigated its consumption of volatile anesthetics at 0.5 MAC (primary endpoint) and the corresponding costs. Secondary endpoints were the technical feasibility to reach and control the MAC automatically, the depth of sedation at 0.5 MAC, and awakening times. Mechanically ventilated and sedated patients after major surgery were enrolled. Upon arrival in the intensive care unit, patients obtained intravenous propofol sedation for at least 1 h to collect ventilation and blood gas parameters, before they were switched to inhalational sedation using MIRUS™ with isoflurane, sevoflurane, or desflurane. After a minimum of 2 h, inhalational sedation was stopped, and awakening times were recorded. A multivariate electroencephalogram and the Richmond Agitation Sedation Scale (RASS) were used to assess the depth of sedation. Vital signs, ventilation parameters, gas consumption, MAC, and expiratory gas concentrations were continuously recorded.

**Results:**

Thirty patients obtained inhalational sedation for 18:08 [14:46–21:34] [median 1st–3rd quartiles] hours. The MAC was 0.58 [0.50–0.64], resulting in a Narcotrend Index of 37.1 [30.9–42.4] and a RASS of − 3.0 [− 4.0 to (− 3.0)]. The median gas consumption was significantly lowest for isoflurane ([ml h^−1^]: isoflurane: 3.97 [3.61–5.70]; sevoflurane: 8.91 [6.32–13.76]; and desflurane: 25.88 [20.38–30.82]; *p* < 0.001). This corresponds to average costs of 0.39 € h^−1^ for isoflurane, 2.14 € h^−1^ for sevoflurane, and 7.54 € h^−1^ for desflurane. Awakening times (eye opening [min]: isoflurane: 9:48 [4:15–20:18]; sevoflurane: 3:45 [0:30–6:30]; desflurane: 2:00 [1:00–6:30]; *p* = 0.043) and time to extubation ([min]: isoflurane: 10:10 [8:00–20:30]; sevoflurane: 7:30 [4:37–14:22]; desflurane: 3:00 [3:00–6:00]; *p* = 0.007) were significantly shortest for desflurane.

**Conclusions:**

A target-controlled, MAC-driven automated application of volatile anesthetics is technically feasible and enables an adequate depth of sedation. Gas consumption was highest for desflurane, which is also the most expensive volatile anesthetic. Although awakening times were shortest, the actual time saving of a few minutes might be negligible for most patients in the intensive care unit. Thus, using desflurane seems not rational from an economic perspective.

*Trial registration* Clinical Trials Registry (ref.: NCT03860129). Registered 24 September 2018—Retrospectively registered.

## Background

Although most national and international guidelines do not recommend the use of volatile anesthetics (VA) such as isoflurane (ISO), sevoflurane (SEVO), and desflurane (DES) for sedation in the intensive care unit (ICU), several studies and current German guidelines state that VA might be a feasible alternative compared to intravenous drugs, especially if fast awakening or a quick extubation after deep sedation is intended [1–4].

The MIRUS™ (TIM, Koblenz, Germany), an electronical gas delivery system introduced in 2013, offers an automated end-expiratory target-controlled application of VA irrespective of the breathing parameters set on the ventilator, which is comparable with the electronic vaporization of some anesthetic machines. This ‘MAC pilot’ is the special feature of the MIRUS™, which consists of a control unit (monitors and controls gas flow, pressure, VA concentration, and VA application), and an ‘exchanger’, which is a VA carbon reflector with filter and heat moisture exchanger. The ‘exchanger’ is inserted between Y-piece of the ICU ventilator and the tracheal tube and connected to the control unit by a special multi-lumen cable. Basically, the MIRUS™ can be used to deliver ISO, SEVO, and DES, but the VA reservoir in the control unit is VA specific [5, 6].

Since cost-effectiveness is of growing interest in ICUs all over the world, the VA consumption and the efficiency of the MIRUS™ is of importance. We defined 0.5 MAC as a target value (age adjusted by MIRUS™) for mechanically ventilated and sedated ICU patients after major surgery and investigated the VA consumption of the system (primary endpoint). Corresponding costs for ISO, SEVO, and DES were calculated. Secondary endpoints were the technical feasibility of the MIRUS™ to reach and maintain the MAC automatically, the depth of sedation at 0.5 MAC judged by the electroencephalography-based Narcotrend^®^ monitor (Narcotrend-Gruppe, Hanover, Germany) and the Richmond Agitation Sedation Scale (RASS), as well as a comparison of awakening times in ISO, SEVO, and DES sedated patients. VA decrement times from 0.5 to 0.25 MAC were additionally recorded.

## Methods

This monocenter randomized controlled trial was performed in a German University Hospital between February 2016 and Mai 2017. It was approved by the appropriate Institutional Review Board (Ethikkommission der Ruhr-Universität Bochum, 4780-13) and registered at the Clinical Trials Registry (ref.: NCT03860129). Written informed consent was obtained from all patients prior to their inclusion in this study. Measurements complied with the ethical standards of the Declaration of Helsinki.

The MIRUS™ systems were kindly provided by the former marketeer (Pall Medical, Dreieich, Germany).

### Study design and setting

We preoperatively enrolled ASA physical status classification I–III patients aged 18–80 years, who were scheduled for major surgery with an expected need for postoperative mechanical ventilation due to respiratory or hemodynamic problems (norepinephrine dose > 0.5 h^−1^, FiO_2_ > 0.5, PEEP > 10 mbar, body temperature < 36 °C). Exclusion criteria were ASA physical status classification IV, malignant hyperthermia, any neuromuscular diseases, increased intracranial pressure, autoimmune hepatitis, pregnancy, deafness, language barriers, involvement in other studies, refusal to give informed consent, and an expected tidal volume < 350 ml.

The closed envelop method was used to allocate patients at random to group ISO, SEVO, or DES.

General anesthesia was induced in the operating room with 0.2 µg kg^−1^ sufentanil and 2 mg kg^−1^ propofol to facilitate intubation with a cuffed tracheal tube. An epidural analgesia (ropivacaine 2 mg ml^−1^) was offered to patients without contraindications. Maintenance of anesthesia was with SEVO (1.0 MAC) and sufentanil. At the end of surgery, the SEVO application was stopped and patients obtained propofol (5 mg kg^−1^ h^−1^) for the transport from the operating room to the ICU.

Upon arrival in the ICU, patients were equipped with a multivariate electroencephalogram (Narcotrend^®^) to assess the depth of sedation. The RASS was additionally gathered by the nurses. After at least 1 h of propofol sedation (2.5 mg kg^−1^ h^−1^), patients were switched to VA sedation via MIRUS™. The patient’s expiratory VA concentration was monitored by MIRUS™ and automatically adjusted to maintain the target value 0.5 MAC. Sufentanil was administered by a syringe pump (5–30 µg h^−1^). The minimum VA sedation time was 2 h.

Lung protective mechanical ventilation (tidal volume 6–8 ml kg^−1^) was performed with a Puritan Bennett 840 ventilator (Covidien, Boulder, Co, USA) on a Bi-Level mode, which allows pressure control and pressure support ventilation. The pressure control mode was solely used until the criteria for a wake-up test were met (norepinephrine < 0.5 h^−1^, FiO_2_ < 0.5, PEEP < 10 mbar, body temperature > 36 °C). Then, the respiratory rate was halved, the pressure support mode used, and a spontaneous breathing trail performed (support 5–15 mbar, PEEP 5 mbar, FiO_2_ 0.3). VA was still administered at 0.5 MAC. The sufentanil dose was not changed initially but reduced by 10 µg kg^−1^ h^−1^ if the patient did not start breathing within 30 min. An end-expiratory CO_2_ < 35 mmHg or > 14 breaths min^−1^ were treated with 5-µg sufentanil. As soon as patients breathed spontaneously (support 5 mbar), the rapid shallow breathing index was calculated (respiratory frequency divided by tidal volume). Once the index was < 105, the MAC was set to 0 with the exchanger within the breathing circuit (start of measuring awakening times). Decrement times from 0.5 to 0.25 MAC were recorded. The rapid shallow breathing index was again calculated immediately before extubation.

RASS was documented twice during propofol sedation, 5, 30, and 60 min after beginning of VA sedation, 60 min before the end and at the end of VA application, as well as after 5, 30, and 60 min in the post-sedation phase.

The Narcotrend^®^ Index (NI), ventilation parameters, VA consumption, MAC, and expiratory VA concentration were continuously provided through the devices. The MAC fraction was evaluated using the formulas MAC_1_ = 1.47 vol% − (age·0.0071 vol%) for ISO, MAC_1_ = 2.31 vol% − (age·0.0106 vol%) for SEVO, and MAC_1_ = 8.43 vol% − (age·0.0428 vol%) for DES according to the manufacturer [7].

Blood gas analyses were performed upon ICU arrival, twice during VA sedation, and after the end of VA application to allow early adjustments of the set ventilator parameters.

**Fig. 1:**
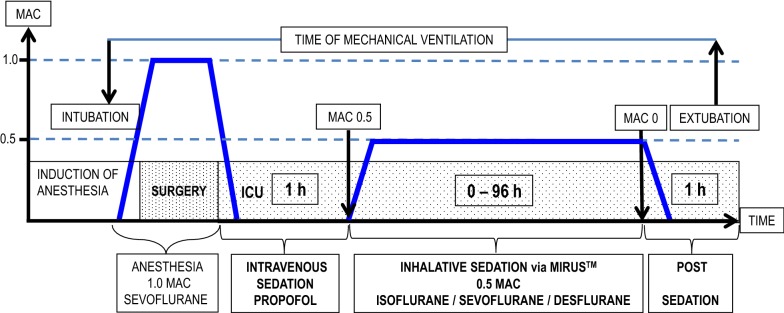
Study design. After intravenous induction of anesthesia in the operating room, patients obtained a balanced anesthesia with sufentanil and sevoflurane at 1.0 MAC via a standard anesthetic machine during surgery. At the end of surgery, sedation was switched to an intravenous regime with propofol for the transport from the operating room to the intensive care unit (ICU). After a minimum of 1-h intravenous sedation in the ICU, patients obtained isoflurane, sevoflurane, or desflurane at 0.5 MAC via MIRUS™. As soon as the criteria for a wake-up test were met, a spontaneous breathing trail was performed. Once patients passed the test the MAC was set to 0. The dark blue lines mark the course of the MAC throughout the study

A summary of the study design is pictured in Fig. 1.

### Sample size calculation

Sample size calculation was conducted in g power (Heinrich-Heine-University, Dusseldorf, Germany) and is based on a pilot study investigating the VA consumption of MIRUS™ at 1.0 MAC during surgery [8]. Significantly different VA consumption rates of the three gases were reported with an estimated effect size of *d* = 3.56, which was transferred to the current study design. For an alpha of 0.05 and a power of 0.8, at least 3 patients per group were needed when analyzed via a univariate ANOVA. We enrolled 10 patients per group to detect effect sizes of at least *d* = 1.2, which displays a strong effect.

### Statistical analysis

All variables were tested for normal distribution using the Kolmogorov–Smirnov test and each variables histogram. A non-parametric approach was chosen to analyze data consistently across groups and variables. All metric variables, except age, height and weight, which are displayed as mean ± 1 standard deviation, were presented as median [1st–3rd quartiles].

Differences across the 3 groups were tested for continuous variables with the Kruskal–Wallis test, which was further explored in case of a significant main effect ‘gas’ with the Mann–Whitney test. Age, height and weight were analyzed via a univariate ANOVA and further investigated with independent *t*-tests. Group-independent comparisons between propofol sedation and VA sedation were analyzed with the Wilcoxon test. Nominal data were analyzed via cross tables and the Chi^2^-test, or with Fishers exact test.

Effect sizes were calculated in case of significant results, meaning for direct group comparisons (Mann–Whitney tests) and for phase comparisons (Wilcoxon test). This was done via $r = z/\sqrt {\text{Number of obersations}}$ with *r* = 0.1 (small effect), *r* = 0.3 (medium effect), and *r* = 0.5 (strong effect) [9].

## Results

A total of 293 patients underwent major surgeries with expected ICU stay during the study period; 78 of these met the inclusion criteria. Forty-eight patients were already extubated in the operating room, whereas the remaining 30 patients (10 per group) completed the study. Their characteristics are shown in Table 1.

**Table 1:** Patient characteristics

	Isoflurane [*n* = 10]	Sevoflurane [*n* = 10]	Desflurane [*n* = 10]	All [*n* = 30]	Statistics
Demography					
Sex (♂/♀) [*n*]	8/2	7/3	5/5	20/10	*p* = 0.500
Age [years]	65.2 ± 9.8	68.4 ± .9.6	59.9 ± 13.29	64.5 ± 11.2	*p* = 0.238
Height [cm]	176.5 ± 8.5	176.6 ± 7.3	167.8 ± 110.7	173.6 ± 9.6	*p* = 0.057
Weight [kg]	84.7 ± 10.2	83.2 ± 20.8	68.7 ± 15.7	78.9 ± 17.2	*p* = 0.067
ASA [score]	2 [1.75−2]	2 [2]	2 [2]	2 [2]	*p* = 0.484
SAPS [score]	28.5 ± 10.4	33.9 ± 10.4	26.2 ± 9.47	29.53 ± 10.3	*p* = 0.234
Comorbidities [*n*]					
Hypertension	4	7	7	18	*p* = 0.452
Coronary heart disease	2	3	0	5	*p* = 0.321
PAOD	0	1	1	2	*p* > 0.999
Nicotine	4	4	2	10	*p* = 0.698
COPD	1	0	1	2	*p* > 0.999
Renal insufficiency	1	1	0	2	*p* > 0.999
Diabetes	3	3	3	9	*p* > 0.999
Alcohol abuse	0	1	4	5	*p* = 0.094
Previous opioid medication	0	0	3	3	*p* = 0.089
Surgeries [*n*]					
Aortic surgery	1	1	3	5	*p* = 0.574
Pancreatic surgery	2	0	3	5	*p* = 0.321
Esophagectomy	5	4	2	11	*p* = 0.321
HIPEC	1	2	1	4	*p* > 0.999
Necrotizing fasciitis	0	1	0	1	*p* > 0.999
Spinal fusion	1	2	1	4	*p* > 0.999
Epidural analgesia [*n*]	3	2	3	8	*p* > 0.999

Demographics, comorbidities, the kind of surgeries, and the number of patients requiring epidural analgesia were comparable throughout all groups. Values are given as mean ± SD, median [1st–3rd quartiles], or number

*PAOD* peripheral arterial occlusive disease, *COPD* chronic obstructive pulmonary disease, *HIPEC* hyperthermic intraperitoneal chemotherapy

### Operating room

Surgery and general anesthesia lasted 5:40 [3:57–7:03] h and 6:27 [4:47–8:18] h (both median; 1st–3rd quartiles), respectively, with no significant difference between the groups (surgery: *p* = 0.312; anesthesia: *p* = 0.704).

During surgery, all patients obtained SEVO at 1.12 [1.02–1.20] MAC and 0.17 [0.12–0.22] µg kg^−1^ h^−1^ sufentanil (median [1st–3rd quartiles]) without statistically significant group differences (MAC: *p* = 0.124; sufentanil: *p* = 0.059).

### Intensive care unit

Upon arrival in the ICU, patients obtained propofol sedation for 1:27 [0:59–2:16] h and VA sedation at 0.5 MAC for 18:08 [14:46–21:34] h (both median, 1st–3rd quartiles). Differences between the three groups were not observed (propofol: *p* = 0.876; VA: *p* = 0.716). Corresponding ventilation and blood gas parameters are shown in Table 2.

**Table 2:** Ventilation parameters and blood gas analysis during postoperative intravenous and VA sedation

	Intravenous propofol sedation					Inhalational VA sedation					IV vs. VA
	Gr. ISO [*n* = 10]	Gr. SEVO [*n* = 10]	Gr. DES [*n* = 10]	All [*n* = 30]	*p* value	ISO [*n* = 10]	SEVO [*n* = 10]	DES [*n* = 10]	All [*n* = 30]	*p* value	*p* value
Ventilation											
Resp. rate [*n*]	14.7 [13.2 to 15.5]	13.9 [12.0 to 15.5]	14.1 [13.8 to 16.1]	14.1 [13.7 to 15.5]	0.588	15.3 [14.3 to 17.8]	14.0 [13.3 to 16.0]	15.1 [14.2 to 18.0]	15.1 [13.9 to 16.9]	0.128	0.065
Tidal vol. [l]	0.58 [0.54 to 0.63]	0.59 [0.54 to 0.64]	0.54 [0.49 to 0.62]	0.59 [0.52 to 0.62]	0.447	0.57 [0.49 to 0.64]	0.62 [0.58 to 0.65]	0.53 [0.48 to 0.63]	0.59 [0.50 to 0.63]	0.385	0.701
RMV [l min^−1^]	8.6 [7.3 to 9.2]	8.3 [7.4 to 9.0]	8.4 [7.2 to 8.8]	8.4 [7.4 to 9.0]	0.907	9.2 [7.5 to 10.2]	9.1 [7.7 to 9.5]	9.1 [7.4 to 9.6]	9.2 [7.6 to 9.8]	0.804	*0.033* **(***r* = 0.275)
etCO_2_ [mmHg]	37.9 [34.09 to 38.73]	34.68 [32.68 to 37.56]	35.94 [32.87 to 39.29]	35.98 [33.13 to 38.72]	0.588	38.0 [37.0 to 40.4]	38.7 [32.5 to 41.9]	37.2 [35.5 to 42.1]	38.0 [35.7 to 41.4]	0.983	*0.041* (*r* = 0.263)
*P*_max_ [mbar]	20.6 [19.1 to 22.6]	17.9 [14.4 to 20.0]	19.0 [16.4 to 22.3]	19.2 [16.5 to 21.5]	0.069	21.8 [19.7 to 24.4]	18.2 [13.5 to 23.8]	22.1 [20.3 to 23.3]	21.5 [18.3 to 23.8]	0.195	0.056
PEEP [mbar]	5.8 [5.6 to 7.6]	6.9 [5.2 to 8.6]	5.5 [5.0 to 5.8]	5.7 [5.5 to 7.4]	0.086	6.9 [5.7 to 8.0]	5.8 [3.7 to 8.0]	6.2 [5.2 to 7.4]	6.7 [5.6 to 7.9]	0.460	0.488
BGA											
pH	7.38 [7.33 to 7.42]	7.38 [7.34 to 7.42]	7.39 [7.36 to 7.44]	7.38 [7.35 to 7.43]	0.854	7.45 [7.42 to 7.50]	7.40 [7.39 to 7.42]	7.46 [7.39 to 7.47]	7.43 [7.39 to 7.47]	*0.037* ^*1^	*0.002* *r* = 0.396
						*^1^ ISO vs. DES: *p* = 0.971; SEVO vs. DES: *p* = 0.052; ISO vs. SEVO: *p* *= 0.011*					
BE [mmol l^−1^]	1.00 [− 1.30 to 2.68]	0.95 [− 0.73 to 1.88]	2.10 [0.28 to 3.90]	1.45 [− 0.25 to 2.68]	0.452	6.20 [2.53 to 6.75]	4.45 [1.88 to 5.95]	6.00 [4.88 to 9.93]	5.60 [3.23 to 6.73]	0.168	*< 0.001* *r* = 0.572

Ventilation parameters were comparable throughout all groups (Gr.) during propofol and VA sedation. However, the respiratory minute volume (RMV) and the end-tidal expiratory CO_2_ concentration increased during VA sedation compared to intravenous sedation. Intra- and inter-group comparisons of blood gas analysis (BGA) parameters were not significantly different for hemoglobin (propofol: *p* = 0.453, VA: *p* = 0.148; IV vs. VA: *p* = 0.105), potassium (propofol: *p* = 0.392, VA: *p* = 0.309; IV vs. VA: *p* = 0.628), natrium (propofol: *p* = 0.402, VA: *p* = 0.085; IV vs. VA: *p* = 0.153), and chloride (propofol: *p* = 0.568, VA: *p* = 0.361; IV vs. VA: *p* = 0.830); pH and base excess (BE) increased during VA sedation compared to intravenous sedation. Intragroup comparisons revealed the lowest pH for SEVO during VA sedation

*Resp. rate* respiratory rate, *vol.* volume

The actual MAC, the corresponding Narcotrend Index as well as the RASS are shown in Table 3.

**Table 3:** Sedative anesthetics and depth of sedation

	Intravenous propofol sedation					Inhalational VA sedation					IV vs. VA
	Gr. ISO [*n* = 10]	Gr. SEVO [*n* = 10]	Gr. DES [*n* = 10]	All [*n* = 30]	*p* value	ISO [*n* = 10]	SEVO [*n* = 10]	DES [*n* = 10]	All [*n* = 30]	*p* value	*p* value
Duration of sedation											
Propofol [h:min]	1:20 [1:00 to 3:14]	1:26 [1:00 to 1:45]	1:30 [1:00 to 4:27]	1:27 [1:00 to 2:16]	0.876	–	–	–	–		
VA [h:min]	–	–	–	–		17:52 [16:37 to 20:38]	16:31 [10:26 to 37:26]	18:52 [14:9 to 33:48]	18:08 [14:46 to 21:34]	0.716	
Sedatives and analgesics											
Propofol [mg kg^−1^ h^−1^]	2.75 [2.14 to 3.09]	2.79 [1.78 to 3.36]	3.25 [2.91 to 4.20]	2.98 [2.32 to 3.33]	0.098	–	–	–	–		
VA [MAC]	–	–	–	–		0.58 [0.56 to 0.59]	0.54 [0.50 to 0.60]	0. 56 [0.53 to 0.60]	0.58 [0.53 to 0.59]	0.326	
etVA [vol. %]	–	–	–	–		0.57 [0.55 to 0.63]	0.84 [0.78 to 1.00]	3.21 [3.04 to 3.69]	0.84 [0.61 to 3.09]	*< 0.001* ^*1^	
						^*1^: ISO vs. DES: *p* *< 0.001*, *r* = 0.84; SEVO vs. DES: *p* *< 0.001*, *r* = 0.84; ISO vs. SEVO: *p* *< 0.001*, *r* = 0.84					
Sufentanil [µg kg^−1^ h^−1^]	0.22 [0.12 to 0.26]	0.20 [0.16 to 0.29]	0.16 [0.14 to 0.33]	0.20 [0.14 to 0.28]	0.938	0.13 [0.08 to 0.21]	0.13 [0.05 to 0.20]	0.22 [0.15 to 0.34]	0.16 [0.10 to 0.23]	*0.024* ^*2^	0.104
						^*2:^ ISO vs. DES: *p* *= 0.035*, *r* = 0.47; SEVO vs. DES: *p* *= 0.011, r* = 0.55; ISO vs. SEVO: *p* = 0.579					
Depth of sedation											
Narcotrend Index [score]	32.4 [26.6 to 41.6]	32.1 [25.6 to 55.2]	40.5 [31.0 to 59.8]	33.1 [28.22 to 49.85]	0.403	33.0 [28.23 to 44.67]	37.3 [29.77 to 44.93]	37.9 [36.53 to 42.12]	37.1 [30.98 to 42.36]	0.665	0.775
RASS [score]	− 5.0 [− 5.0 to (− 4.8)]	− 5.0 [− 5.0 to (− 5.0)]	− 4.8 [− 5.0 to (− 3.0)]	− 5.0 [− 5.0 to (− 4.4)]	0.165	− 3.0 [− 4.1 to (− 3.0)]	− 3.3 [− 4.3 to (− 3.0)]	− 3.0 [− 3.6 to (− 2.8)]	− 3.0 [− 4.0 to (− 3.0)]	0.410	*< 0.001* *r* = 0.52

A MAC of 0.58 and a propofol infusion rate of 2.98 ml kg^−1^ h^−1^ led to a comparable depth of sedation as measured by Narcotrend Index. According to RASS, nurses judged VA sedated patients more ‘awake’ than propofol sedated patients. Values are given as median [1st–3rd quartiles]

*Gr.* group

The median VA consumption [ml h^−1^; 1st–3rd quartiles] was 3.97 [3.61–5.70] for ISO, 8.91 [6.32–13.76] for SEVO, and 25.88 [20.38–30.82] for DES (all: *p* < 0.001; ISO vs. DES: *p* < 0.001, *r* = 0.845; SEVO vs. DES: *p* < 0.001, *r* = 0.761; ISO vs. SEVO: *p* = 0.004, *r* = 0.625).

The consumption corresponds to average costs of 0.39 € h^−1^ for ISO, 2.14 € h^−1^ for SEVO, and 7.54 € h^−1^ for DES.

**Fig. 2:**
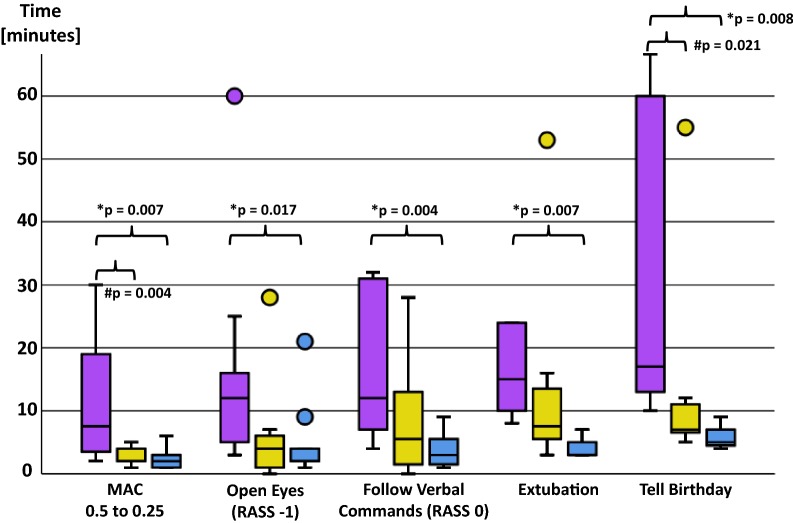
Awakening times. The time needed to decrease the MAC from 0.5 to 0.25 was longest for ISO (pink box) and quickest for DES (blue box). Correspondingly, awakening was quickest after DES sedation, followed SEVO (yellow box) and ISO. Median (horizontal black lines), 1st and 3rd quantile (upper and lower end of the boxes), the 95% interval (horizontal black lines) and statistical outliers (circles: outside the 95% interval) are presented. *DES is significantly quicker than ISO; ^#^SEVO is significantly quicker than ISO

Awakening and MAC decreasing times are shown in Fig. 2.

## Discussion

In this study, we investigated the VA consumption of the MIRUS™ System at a target value of 0.5 MAC, its technical feasibility to reach and maintain the MAC, corresponding depths of sedation, and awakening times in 30 patients after major surgery in the ICU. Data revealed that an approximately twofold larger amount of SEVO, and a sixfold larger amount of DES compared to ISO are needed to achieve a comparable depth of sedation at 0.5 MAC (judged by NI and RASS). However, recovery times were the longest when using ISO.

Various studies have shown that VAs may be beneficial for sedation of critically ill patients in the ICU because of their minimal systemic metabolism rate, organ protective effects, the rare occurrence of tolerance or ceiling effects, the possibility to control the effective concentration by monitoring the end-tidal VA concentration, as well as due to their rapid onset and offset of action with fast emergence, easy weaning, and extubation [6, 10–14]. However, most physicians still prefer a sedation practice based on intravenous agents. Besides structural (e.g., no air-conditioning) or medical reasons for it, two frequently mentioned concerns about VA sedation in the ICU are the high costs and possible problems during mechanical ventilation due to the device’s dead space of approximately 100 ml.

Indeed, the application of VA by MIRUS™ in the ICU requires technical prerequisites and is, therefore, costly. The VA-specific control unit is the most expansive part of the MIRUS™ System. However, it is not disposable material. The exchanger, which consists of the reflector with HME filter, must be replaced once a week. It costs about 150 € or 1 € h^−1^. Its long durability is achieved by the fact that the filter is kept separate from the carbon reflector [15]. Furthermore, devices to reduce atmospheric VA pollution must be considered. Studies have shown that an air-conditioning with at least six air changes per hour and a scavenging system should be used to reach mean VA concentrations below 1 ppm in the patient room (the National Institute of Occupational Safety and Health has defined an exposure limit of 2 ppm for ISO, SEVO, and DES in the air) [16, 17].

The VA consumption is of special interest, since it mainly influences the running costs. The hourly consumptions in this study were approximately 4 ml for ISO, 9 ml for SEVO, and 26 ml for DES to reach 0.5 MAC. ISO, the cheapest VA, costs 0.39 € h^−1^; propofol, in comparison, about 0.41 € h^−1^ for an infusion rate of 3 mg kg^−1^ h^−1^. Although comparable studies are rare, Romagnoli and colleagues reported nearly identical results for SEVO of approximately 8 ml for a slightly lower MAC of 0.45 [17]. Another observation, a case report of an obese woman suffering from acute respiratory distress syndrome after aspiration of gastric contents, demonstrated a twofold higher VA consumption of 53 ml h^−1^ DES to reach a fraction of 3.3–3.8% (3.21% in this study) [18]. However, it must be noted that the VA consumption depends (besides the MAC) on the loss of VA through the reflector, which is influenced by the respiratory minute volume [19, 20]. Thus, the respiratory minute volume was actually lower in the study of Romagnoli et al. (7.5 l min^−1^), and higher in the case report (up to 12 l min^−1^) than in this study (9 l min^−1^).

Two studies investigated the VA consumption of MIRUS™ at 1.0 MAC. A benchmark study reported a DES consumption of 40 ml per hour to achieve a fraction of 6.0–6.6%, and a clinical trial revealed a threefold higher consumption for ISO and SEVO, as well as a one and a half times higher consumption for DES compared to our results during hip and knee replacement surgery (respiratory minute volume 6–7 l min^−1^) [5, 8]. These high consumptions can be explained by the fact that the reflection efficiency of the MIRUS™ is highest at expiratory VA fractions of about 0.2–1.0%, and thus, more VA gets lost at higher concentrations [5].

Rebreathing and hypercarbia could be adverse effects when using the MIRUS™ because of its dead space. Data demonstrated that etCO_2_ increases significantly during VA sedation compared to intravenous sedation, although the respiratory minute volume had been raised by 0.8 l min^−1^. However, on the one hand, the pH was still within a normal range (it even increased), and on the other hand a lot of different factors could have caused these changes.

Awakening times and mental recovery were significantly shortest after DES sedation, although patients in this group obtained the highest amount of sufentanil. Moreover, all patients opened their eyes within 10 min after VA application had stopped, even though sedation lasted 18 h on average and we did not remove the reflector (it is to be expected that VA concentrations decrease more quickly without reflector; however, we did not remove it to monitor the VA concentration, pressure and flow, as well as to collect these data automatically for later analysis). This quick awakening is consistent with previous observations and can be attributed to the VAs’ characteristics (quick washout times, small risk of oversedation due to real-time feedback, low metabolic rates) [4, 21]. Furthermore, it is of advantage since it has been shown that a quick interruption of sedation accompanied by early awakening and extubation, as well as the control of the depth of sedation improves clinical outcomes in postoperative ICU patients [22, 23]. However, DES is not rational from an economic point of view, especially because the time saving of a few minutes compared to ISO is negligible for most ICU patients. Thus, ISO should be preferred.

To our knowledge, this is the first study using an automated target-controlled MAC and not an expiratory VA fraction to perform sedation in the ICU. The target value was reliably reached by MIRUS™, and the NI was comparable in all groups. Furthermore, the MAC was kept constant during hyperventilation and hypoventilation, since it is independent of the respiratory minute volume (no inadequate VA application).

This study lacks a common intravenous control group, which is a limitation. However, we performed case–control-group comparisons within the study population. This approach enabled us to compare ventilation and blood gas parameters, NI, and RASS during intravenous and VA sedation within the same patient. Awakening times could obviously not be determined for propofol; however, the comparison between propofol and VA has already been investigated by others [3, 21]. Another limitation is the study’s small sample size. This investigation was designed to detect strong effects only; however, the described effects must be considered significant. Furthermore, we did not focus on clinical effects or outcome parameters, respectively. This must be elucidated in follow-up studies. Lastly, we focused on a well-selected patient group without severe organ dysfunctions. It remains questionable whether such patients must receive sedation in the postoperative period. Thus, our results must be verified in a broader ICU population of critically ill patients.

## Conclusions

In summary, a target-controlled, MAC-driven automated VA application of 0.5 MAC for sedation in the ICU is technically feasible and enables an adequate depth of sedation. The gas consumption is highest for DES, which is also the most expansive VA, and the actual time savings is only a few minutes, which is clinically insignificant for most ICU patients. Thus, the use of DES seems not rational from an economic perspective. Follow-up studies are needed to elucidate outcome effects of VA sedation in the ICU.

## Data Availability

A limited de-identified dataset is available from the corresponding author on reasonable request.

## Abbreviations

ASA Class: American Society of Anesthesiologists Classification
DES: desflurane
ICU: intensive care unit
ISO: isoflurane
MAC: minimum alveolar concentration
NI: Narcotrend Index
PEEP: positive end-expiratory pressure
RASS: Richmond Agitation Sedation Scale
SEVO: sevoflurane
VA: volatile anesthetic
